# Hypoxia and Inflammation in Cancer, Focus on HIF and NF-κB

**DOI:** 10.3390/biomedicines5020021

**Published:** 2017-05-09

**Authors:** Laura D’Ignazio, Michael Batie, Sonia Rocha

**Affiliations:** Centre for Gene Regulation and Expression, School of Life Sciences, University of Dundee, Dundee DD15EH, UK; l.dignazio@dundee.ac.uk (L.D.); m.t.batie@dundee.ac.uk (M.B.)

**Keywords:** NF-κB, hypoxia, inflammation, κB Kinase (IKK), Prolyl Hydroxylases (PHDs), cancer, Transforming Growth Factor-β-Activated Kinase 1 (TAK1), Factor Inhibiting HIF (FIH)

## Abstract

Cancer is often characterised by the presence of hypoxia and inflammation. Paramount to the mechanisms controlling cellular responses under such stress stimuli, are the transcription factor families of Hypoxia Inducible Factor (HIF) and Nuclear Factor of κ-light-chain-enhancer of activated B cells (NF-κB). Although, a detailed understating of how these transcription factors respond to their cognate stimulus is well established, it is now appreciated that HIF and NF-κB undergo extensive crosstalk, in particular in pathological situations such as cancer. Here, we focus on the current knowledge on how HIF is activated by inflammation and how NF-κB is modulated by hypoxia. We summarise the evidence for the possible mechanism behind this activation and how HIF and NF-κB function impacts cancer, focusing on colorectal, breast and lung cancer. We discuss possible new points of therapeutic intervention aiming to harness the current understanding of the HIF-NF-κB crosstalk.

## 1. NF-κB Subunits and Signalling Pathways

NF-κB is the collective name of a family of transcription factors initially discovered in 1986 by Ranjan Sen and David Baltimore as a Nuclear Factor binding to the enhancer element of the immunoglobulin κ light-chain of activated B cells [1]. Over the years, a crucial role in controlling gene expression in response to inflammation, proliferation, differentiation, among other physiological processes, has been assigned to the five gene members of this protein family: RelA (p65), RelB, c-Rel, NF-κB1 (p105) and NF-κB2 (p100) [2,3]. All NF-κB subunits feature high structural similarity, with the N-terminal harbouring a Rel Homology Domain (RHD). This is essential to mediate DNA binding to κb sites in enhancers/promoters of target genes, as well as to dimerise with other subunits. In fact, formation of homo- and heterodimers determines the specificity of the transcriptional response in accordance with the cellular context [4]. Induction or repression of gene expression is achieved not only through combination of different NF-κB dimers, but also by physical association with co-activators, co-repressors, and other transcription factors, such as Signal Transducer and Activator of Transcription 3 (STAT3), p53 [5], or HIFs [6]. A further control of the transcriptional activity also derives from the C-terminal structure of the NF-κB subunits. In particular, RelA, RelB and c-Rel contain trans-activation domains (TAs), whereas p105 and p100, precursors of their active forms p50 and p52, contain IκB-like Ankyrin repeat (ANK) domains, acting as internal inhibitors. Consequently, p50 and p52 can only function as transcriptional activators in association with other subunits or proteins containing the trans-activation domain [5,7].

**Figure 1:**
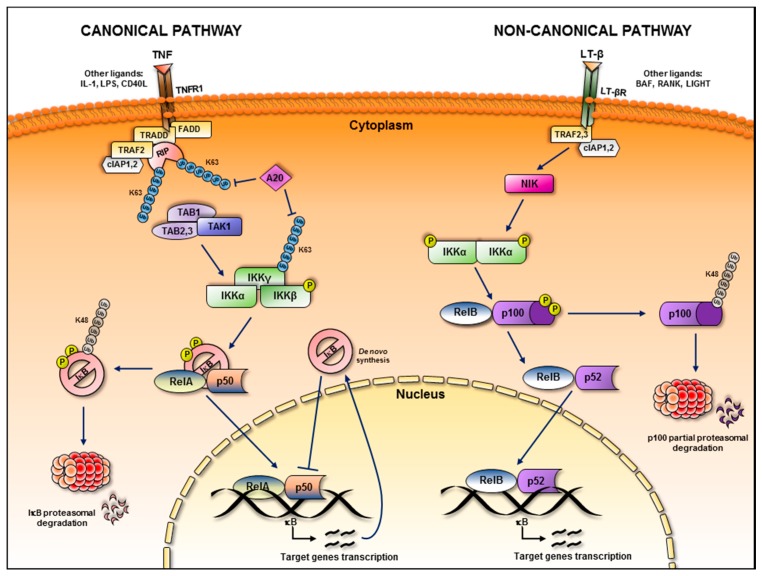
Activation pathways for canonical and non-canonical NF-κB signalling. Canonical NF-κB pathway is exemplified by TNF-α binding to its reception, while non-canonical NF-κB pathway is illustrated by binding of LT-β to its receptor.

Activation of NF-κB can occur following canonical, non-canonical and atypical pathways, all triggered by different stimuli, as elegantly reviewed elsewhere [8,9]. Here, we will briefly focus only on the canonical and non-canonical mechanisms of activation (Figure 1), which are more likely to be involved in malignancies. In the canonical NF-κB signalling pathway, the binding of Tumour Necrosis Factor α (TNF-α), Lipopolysaccharides (LPS), or Interleukin-1 (IL-1) to their specific receptors on the cellular membrane, induces in the cytoplasm the recruitment of several adaptors and protein kinases, serving as activation platform, ultimately leading to the phosphorylation and activation of the Inhibition of κB Kinase (IKK) complex. The IKK complex is formed by two catalytic proteins (IKK1/IKKα and IKK2/IKKβ) and one regulatory protein (IKKγ/NEMO, NF-κB Essential Modulator). An activated IKK complex is then able to phosphorylate the IκB inhibitor molecules, which, in quiescent cells, hold the NF-κB subunits inactive in the cytoplasm. In humans, the most common NF-κB inhibitor protein is IκBα. Phosphorylation of IκBα on serines 32 and 36 is the key prerequisite for the lysine-48 polyubiquitin chain formation catalysed by SCF^βTrCP^ E3 ubiquitin ligase, and subsequent degradation mediated by the proteasomal machinery. This event makes the NF-κB dimers free to translocate into the nucleus, binding the cognate DNA sequence to regulate gene transcription [10]. On the other hand, the non-canonical NF-κB signalling pathway depends on activation of different membrane receptors, such as Lymphotoxin β-Receptor (LTβR), B-cell Activation Factor Receptor (BAFFR), TNF Receptor 2 (TNFR2) and several others. The consequent signal transduction involves the activation of the NF-κB Inducing Kinase (NIK) that, in turns, phosphorylates and activates IKKα homodimers. This event is followed by specific phosphorylation of serines 866 and 870 at the C-terminal region of p100, resembling the phosphorylation site of IκBα. Upon binding of the SCF^βTrCP^ ubiquitin ligase, the inhibitor ankyrin repeat domain of p100 is subject to proteasomal processing. Thus, the cleaved active form p52 originates and associates with RelB, serving as transcriptional activator heterodimer after translocation into the nucleus [7].

In general, the fine tuning of the NF-κB response is controlled by different post-translational modification events, such as phosphorylation, as well as by an intricate series of protein-protein interactions and feedback loops. The fact that IκBα itself is one of the NF-κB target genes represents an example of negative feedback. The newly synthesized IκBα is able to relocate NF-κB subunits from the nucleus to the cytoplasm. Also, considering the important role of K63-linked or linear polyubiquitination as a platform for the activation of the NF-κB pathway, de-ubiquitinase (DUB) enzymes, such as Cyld or A20, can serve as negative feedback inhibitors of the pathways [5]. When the regulatory mechanisms described above become aberrant, NF-κB can become constitutively active or deregulated. For instance, NF-κB is chronically activated in several inflammatory diseases, such as arthritis, inflammatory bowel disease, asthma and many other pathological conditions, including cancers [11,12]. Here, we will discuss the role of NF-κB in cancer, focusing on how this transcription factor can be induced by hypoxia, and modulated by HIF, in a variety of tumour contexts.

## 2. NF-κB in Inflammation and Cancer

Activation of the NF-κB pathway is widely recognised as characteristic of inflammation. Inflammation is a defensive process used by the innate and adaptive immune systems to respond to bacterial and viral infections, facilitating wound healing or maintenance of tissue homeostasis. In the last fifteen years, chronically prolonged inflammatory response has been identified as a hallmark of cancer [13]. However, the role of NF-κB in malignancies remains quite controversial, acting as tumour promotor or tumour suppressor depending on the cellular context [14]. In cancers featuring a chronic inflammatory microenvironment, NF-κB is conspicuously activated. Thus, cancer development is further promoted, and tumour progression is reinforced. In fact, in these tissues, pro-inflammatory cytokines (i.e., TNF-α, IL-1, interleukin-6 (IL-6)), Reactive Oxygen and Nitrogen Species (RONS), prostaglandins, and microRNAs accumulate, contributing to create a pro-tumorigenic microenvironment. In particular, a constitutive activated NF-κB participates in carcinogenesis stimulating cell proliferation, inhibiting programmed cell death, regulating angiogenesis, promoting tumour metastasis and remodelling tumour metabolism [14]. NF-κB influences cell proliferation by controlling autocrine and paracrine production of cytokines, such as Interleukin-2 (IL-2) and Granulocyte-Macrophage Colony-Stimulating Factor (GM-CSF) [15]. Additionally, NF-κB controls gene expression of G1 cyclins, such as cyclin D1 [16], which is a crucial protein in the exit from the G1 phase of the cell cycle, frequently altered in numerous human cancers [17].

NF-κB plays a dual role in controlling apoptosis. Several reports have supported a pro-apoptotic activity of NF-κB [18,19]. However, NF-κB ability to prevent apoptosis seems to be more often studied, leading to the activation of target genes such as cellular Inhibitors of Apoptosis (cIAP1/2, XIAP), cellular FLICE (Caspase8/FADD-like IL-1β-Converting Enzyme)-Inhibitory Protein (c-FLIP), and members of the Bcl2 family, such as Bcl-xL [20]. Such anti-apoptotic roles of NF-κB might be determinant during cancer progression, when cells that have undergone DNA damage or chromosomal rearrangements can therefore escape from apoptosis, overcoming also other checkpoint controls normally operated by p53, a tumour suppressor mutual antagonist of NF-κB [21].

Expansion of vascular network, also termed angiogenesis, is one of the main hallmarks of tumour growth [13]. A number of NF-κB target genes are known to be involved in this process. Among them there are the chemokine Interleukin-8 (IL-8) [22], and the Vascular Endothelial Growth Factor (VEGF) [23]. Anti VEGF strategies have been successful in the clinic for treatment of patients with several cancers, including colorectal cancer (CRC) (reviewed in [24]), in the form of Bevacizumab, a monoclonal antibody against VEGFA, and Aflibercept, a recombinant fusion protein blocking VEGFA and VEGFB signalling. Interestingly, these genes can be also targets of HIF-1α [25,26], highlighting the existence of an intricate crosstalk between inflammation and hypoxia in cancer cells (see Section 4).

NF-κB also directly regulates expression of genes encoding for matrix metalloproteinases, such as MMP-9 [27]. Extracellular matrix remodelling not only facilitates the spread of endothelial cells (angiogenesis), but also of cancer cells (metastasis) in the surrounding areas. Notably, a role of NF-κB in tumour metastasis formation has been reported in human head and neck squamous cell carcinoma [28], and breast cancer [29].

As part of the immune response, NF-κB can exert also tumour suppressing functions. In particular, this occurs in some acute inflamed environments, when Cytotoxic T cells (CTL) are highly activated against malignant cells [30], or in chemically induced liver and skin cancers [31,32]. However, this response might be not able to eradicate all aberrant cells, which then escape the immune system. Chronic inflammation in the microenvironment, and, consequently, the increased cytokine release elevates the NF-κB activity, resulting in tumour promotion. However, the NF-κB activity can be enhanced also by mutation of NF-κB pathway components and/or oncogenes [33]. Mutation of NF-κB occurs in particular in lymphoid malignancies, such as human B cell- [34,35] or T cell-lymphomas [36]. Although less frequently, mutations of the NF-κB signalling pathway occur also in solid tumours. For instance, mutations in NF-κB1 have been detected in breast cancer [37]. In addition, most recently, high expression of IKKα has been associated with poor outcome in patients with Estrogen Receptor (ER)-positive invasive ductal breast cancer, although its expression appeared to be independent of NIK and RelB [38]. Overall, the NF-κB pathway has been found to be fundamental in development, maintenance, or invasiveness of multiple solid cancer types, including colon and lung cancers [39,40,41], hepatocellular carcinoma [42], and melanoma [43]. While not all cancers form in inflamed tissues, inflammation arises as a consequence of metabolic dysregulation associated with tumour development, facilitating the progression of the tumour itself. During tumour propagation, the high demand of oxygen and nutrients creates a pathologic hypoxic microenvironment in the tumour core, inducing the production of angiogenic growth factors and cytokines, to form new blood vessels and recruit more innate immune cells [44]. Indeed, hypoxia strongly impacts on tumour progression and metastasis, by activating specific transcriptional programmes, such as HIF and NF-κB [45].

## 3. Hypoxia and Hypoxia Inducible Factor (HIF) Pathway

Hypoxia, or diminished oxygen availability, is a common feature of the tumour microenvironment, where the oxygen level is often below 1%. This condition triggers a series of gene expression changes, affecting angiogenesis and metabolism, to enable tumour survival and progression [45]. Hypoxia in tumours occurs in different areas, from anoxic regions further away from the blood vessels to hypoxic areas, generated by highly metabolic cancer and immune cells [45,46], creating a non-uniform tumour microenvironment. The main molecular drivers of this response belong to a family of basic Helix-Loop-Helix-Per-ARNT-Sim (bHLH-PAS)-containing transcription factors, known as HIFs [47]. HIF is a heterodimeric complex formed by an oxygen-dependent α subunit and an oxygen-insensitive β subunit. In fact, the constitutively expressed HIF-1β (also called ARNT, Aryl Hydrocarbon Receptor Nuclear Translocator) is the obligatory binding partner for any HIF-α. So far, three α-forms (HIF-1α, -2α and -3α) have been identified in humans. HIF-1α, the most well-studied isoform, is ubiquitously expressed, while HIF-2α expression is restricted to endothelial cells, heart, lung, placenta and kidney [48]. Only recently the expression profile of the several HIF-3α spliced variants has been elucidated, with HIF-3α predominantly expressed in kidney and lung epithelial cells [49]. Interestingly, although HIF-1α and HIF-2α are structurally closely related, and despite some redundancy in their functions with the sharing of several common target genes, they also have distinct target gene cohorts [50]. Moreover, their roles can vary depending on tumour and cell types [51]. HIF-3α function remained unclear for long time, being mainly considered a dominant negative regulator of the other HIF-α isoforms, by competing for HIF-1β binding [50]. Only recently, a novel transactivation activity induced by hypoxia has been attributed to HIF-3α in zebrafish embryos, opening new scenarios in the regulation of transcriptional response following exposure to low oxygen [52].

**Figure 2:**
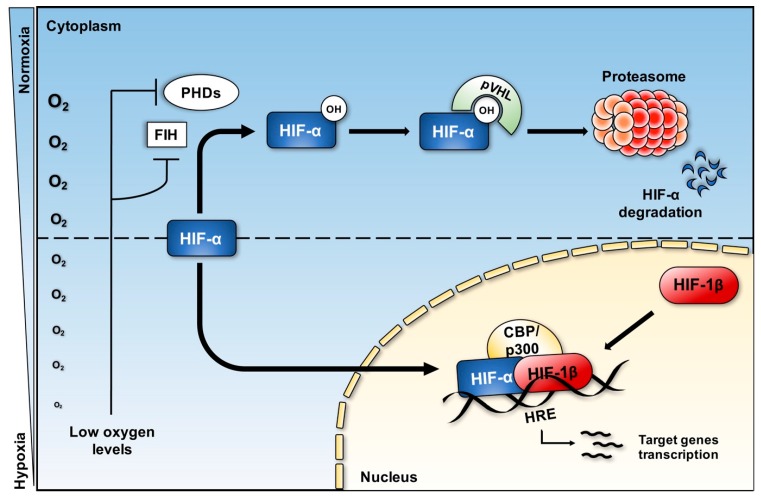
Activation of the Hypoxia Inducible Factor (HIF) pathway in response to hypoxia. HIF-α levels are controlled in normoxia by PHD-mediated hydroxylation (OH) and recognition by the E3-ligase complex containing the tumour suppressor pVHL. In hypoxia, Prolyl Hydroxylases (PHDs) and FIH are inhibited and HIF-α escapes VHL-dependent degradation. T-bars represent repression.

The cellular response to hypoxia engages HIF primarily at the post-transcriptional level, where the HIF-α proteins stability is regulated by non-heme, Fe^2+^ and 2-oxoglutarate (2OG)-dependent dioxygenase enzymes called Prolyl Hydroxylases (PHDs). Under normal oxygen conditions, PHDs (PHD1, PHD2 and PHD3) hydroxylate specific proline residues within the Oxygen Dependent Domain (ODD) of the HIF-α subunit. Thus, the hydroxylated HIF-α is recognised by the von Hippel-Lindau (vHL) tumour suppressor protein, component of an E3-ubiquitin-ligase complex. This leads to HIF-α Lys48-linked poly-ubiquitination and subsequent proteasomal degradation. In addition, further control of transcriptional activity of HIFs escaping degradation is mediated by FIH (Factor Inhibiting HIF). This 2OG-dependent dioxygenase enzyme prevents HIF association with the co-activators p300/CREB Binding Protein (CBP), by hydroxylation of a key asparagine in the transactivation domain of HIF-α. In hypoxia, PHDs and FIH are inactive, due to the absence of oxygen, essential cofactor for these enzymes [53]. This enables the hypoxic transcriptional programme to occur. The stabilised HIF-α subunit translocates into the nucleus, dimerizes with HIF-1β, and, upon binding to the consensus hypoxia response element (HRE), transactivates downstream target genes, involved in a large variety of processes, including glycolysis, angiogenesis, proliferation, migration, and apoptosis [54] (Figure 2). As crucial mediators of several biological and cellular processes, both HIF-1α and HIF-2α expression are elevated in numerous solid tumours, such as colon, breast and lung cancers [48]. For instance, high levels of HIF-1α correlate with poor clinical outcomes in human breast cancer, with HIF-1α being the master regulator of Epithelial-Mesenchymal Transition (EMT), invasion, extravasation, and metastasis in this type of tumor [55].

A relevant role as regulators of the hypoxic response has been recently attributed to the Jumonji C (JmjC) domain containing proteins [56], many of which are 2-OG dioxygenases functioning as protein demethylases. These enzymes, of which there are over 30 discovered in humans, mainly control histone methylation, and they are often deregulated in many cancers (reviewed in [57]). Structural work from the Schofield and Allshire laboratories has revealed that the domain responsible for demethylase activity of these enzymes (JmjC domain) has a fold that is remarkably similar to the catalytic core of FIH [58,59]. In addition, investigation of the oxygen dependency of two of these enzymes revealed a graded drop in activity over physiologically relevant ranges of oxygen [60,61]. These studies indicate the potential of JmjC enzymes to link chromatin structure to oxygen sensing and participate in the hypoxia mediated transcriptional response. Indeed, an increasing number of reports found elevated histone methylation marks in response to prolonged hypoxia [62,63,64,65], with the impaired JmjC enzyme activity being responsible for these changes [63,65]. Interestingly, many of these enzymes are hypoxia inducible (reviewed in [57]), with some, including Lysine (K)-specific demethylase 4B (KDM4B), KDM4C, KDM5B, KDM3A, KDM2A and KDM2B, being HIF targets [66,67,68,69,70,71]. This may point to negative feedback mechanism to help regulate histone methylation in a JmjC histone demethylase compromised environment, and possibly help the cell reset its oxygen sensing and response mechanisms after prolonged hypoxia and/or restoration of normoxia, as is seen with PHD regulation of HIF.

## 4. Crosstalk between Hypoxia and Inflammation in Cancer

**Figure 3:**
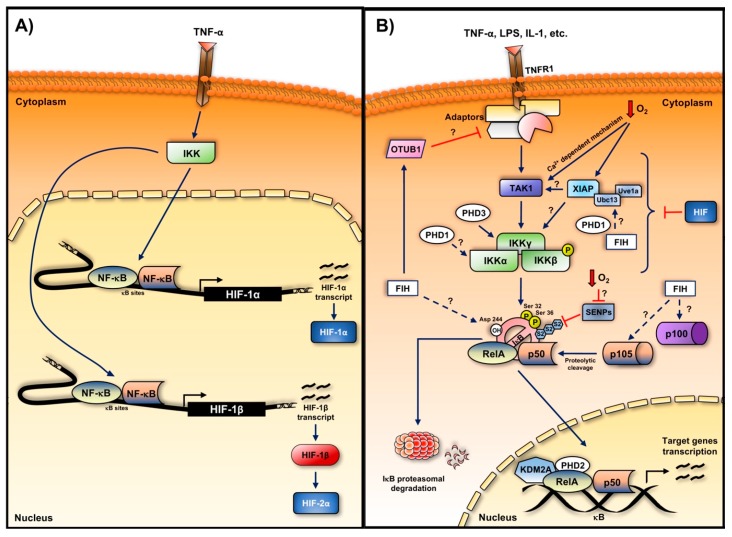
Levels of crosstalk between the HIF and the NF-κB pathways. (**A**) NF-κB control over the HIF pathway; (**B**) Reported points of interaction and control of the HIF pathway over the NF-κB signalling cascade. Red T-bars indicate inhibition points, whereas arrows with dashed lines indicate regulation events that have not been proved in an in vivo system yet. Question marks are used to highlight the unanswered questions into the HIF regulation of the NF-κB pathway.

Recently, an increasing number of studies supported a role of HIF beyond the hypoxia response [72]. HIF-1α activation has been detected following different bacterial infections under normal oxygen levels [73], whereas HIF-2α and HIF-1β have been found to regulate neutrophilic inflammation and myeloid cells function in wound healing, respectively [74,75]. In inflammation, mechanisms leading to HIF induction can be oxygen-independent, and mediated by other transcription factors, such as STAT3 [76] and NF-κB [77,78]. In recent years, an intimate crosstalk between HIF and NF-κB has been appreciated at different levels, as reviewed in [6]. Interestingly, this crosstalk is bi-directional. In fact, not only does NF-κB induce HIF (Figure 3A), but HIF regulates NF-κB (Figure 3B). Particularly, under inflammatory conditions, NF-κB transcriptional activity is restricted by HIF-1α in vivo and in vitro [25]. To date, the knowledge concerning HIF-2α and HIF-1β contribution to the NF-κB activity is still poor, despite the fact that these HIF subunits have been associated with NF-κB [79,80].

HIF can directly contribute to the inflammatory response, inducing several pro-inflammatory chemokines and cytokines [12]. Importantly, numerous genes transcriptionally activated by HIF are also NF-κB target genes involved in tumorigenesis, such as IL-6, MMP9, cyclooxygenase 2 (COX2), as well as pro-survival genes, such as Bcl-2, among others [81]. Indeed, the cooperative relationship between HIF and NF-κB in the tumour-associated inflammation is evident. More pro-inflammatory mediators are produced in the hypoxic areas of the tumours, resulting in the recruitment of more immune cells at neoplastic sites. This determines a chronic inflammation in the tumour, with consequent high activation of NF-κB [12]. As previously stated, inflammation is a key player in tumour development and progression, therefore it is not surprising that chronic inflammatory diseases may predispose to cancer. One example is the Inflammatory Bowel Disease (IBD), a chronic intestinal disorder including Crohn’s disease and ulcerative colitis [11]. Patients affected by this condition have a greater risk to develop colon cancer, particularly Colitis-Associated Colon cancer (CAC) [82]. In colon tumorigenesis, where hypoxic inflammation is significant, both HIF-1α and HIF-2α are expressed [48]. Notably, a recent study highlighted the importance of intestinal epithelial HIF-2α in the recruitment of neutrophils to colon tumour sites, supporting its prominent role in the inflammatory microenvironment [83]. Another example of hypoxia and inflammation crosstalk in cancer is observed in Hepatocellular Carcinoma (HCC), where TNF-α is one of the cytokines constantly activated by NF-κB, through the Tumour Associated Macrophages (TAMs). The expression of HIF-1α and HIF-2α in these immune cells seems to be particularly important in the HCC progression [12,84]. However, to better understand the precise mechanisms, by which hypoxia and immune cells contribute to the intra-tumoural inflamed microenvironment, further studies are needed.

## 5. Hypoxia-Induced NF-κB

As mentioned earlier, NF-κB is one of several transcription factors induced by hypoxia. Although mechanisms by which NF-κB is activated under low oxygen are still under investigation.

### 5.1. Role of the Oxygen Sensors in the Hypoxia Induction of NF-κB

#### 5.1.1. Hydroxylases

2OG-dependent dioxygenases, such as PHDs and FIH, function as oxygen sensors stabilizing HIF in hypoxia, therefore these and other dioxygenases may confer oxygen sensitivity to additional pathways regulated by oxygen availability, including NF-κB. Indeed, the discovery of new and potential FIH and PHD targets supports this. Proteomics approaches have revealed NF-κB pathway components, including p105 and IκBα [85] as well as an upstream regulator of the NF-κB pathway, OTU dDe-ubiquitinase, Ubiquitin Aldehyde Binding 1 (OTUB1), as being hydroxylated by FIH [86,87]. Although these novel FIH targets provide promising links to NF-κB and oxygen sensing, with the exception of OTUB1 [87], mutational analysis has been unsuccessful in identifying any functional significance for these modifications [85,88]. Although OTUB1 hydroxylation by FIH regulates metabolic processes in the cell [87], a role of OTUB1 in activating the NF-κB pathway under hypoxic conditions has yet to be established. Interestingly, other players in the IL-1β pathway, the ubiquitin ligase enzymes Uve1a and Ubc13, are also targets for hydroxylation [86]. These enzymes have also been shown to be required for hypoxia induced NF-κB activity [89,90]. Thus, although speculative, oxygen regulated hydroxylation of these ubiquitin ligases could play a part in the NF-κB induction by hypoxia.

As with FIH, whilst there is evidence of PHD regulation of NF-κB induction following oxygen deprivation, a direct oxygen sensing mechanism mediated by PHD prolyl hydroxylase activity has not been discovered yet. PHDs have been found to antagonise NF-κB activity in various cell types [91,92,93,94,95]. Hypoxia induction of NF-κB via IKK activation has been shown to be regulated by PHD1 and, to a lesser extent, by PHD2 levels [91]. The authors of this study suggested a PHD prolyl hydroxylase dependent mechanism, providing evidence that IKKβ is a potential PHD target through the identification of conserved PHD prolyl hydroxylation motif, which is required for the hypoxia induction of IKKβ levels. Further supporting this finding, Zheng and colleagues identified IKKβ among the PHD1 substrates in a hydroxylation screening assay [96]. However, further studies are required to demonstrate IKK hydroxylation in cells. Conversely, another group has found that PHD3 inhibits NF-κB by a prolyl hydroxylase-independent inhibition of IKKγ ubiquitination [94]. In addition, a cooperative role of PHD2 with respect to NF-κB activity, functioning as coactivator of p65, has been shown [97]. Taken together, these studies exemplify the cell type and context specificity of hypoxia induced NF-κB regulation by PHDs.

#### 5.1.2. JmjCs

The aforementioned JmjC enzymes also link NF-κB activities’ in hypoxia to oxygen sensing. Despite methylated histone lysine residues being the prominent target for the demethylating activity of these enzymes, non-histone targets are emerging. KDM2A has been shown to demethylate p65, inhibiting expression of some of its target genes [98,99]. Various methylation sites have been identified on p65 [98,100,101,102]. Both K218 and K221 methylation sites are reversibly regulated by Nuclear Receptor Binding SET Domain Protein 1 (NSD1) and KDM2A, with NSD1 methylating them in response to IL-1β induction, aiding the activation of a subset of NF-κB target genes. Interestingly, KDM2A is NF-κB inducible [98] as well as hypoxia inducible in HIF-1 dependent manner, as recently shown by our group [71]. This represents another feedback loop of NF-κB regulating its own activity, and may confer another level of crosstalk between low oxygen availability and NF-κB function. KDM2B, the other member of KDM2 family member, is also hypoxia and NF-κB inducible [71,103]; however, it is currently unknown if this enzyme influences NF-κB activity. Another NF-κB induced JmjC enzyme, KDM6B, promotes activation of a subset of genes in LPS activated macrophages in a histone demethylase independent manner [104]. Furthermore, JMJD8 has been shown to positively regulate TNF induced NF-κB signalling, although the mechanism by which this occurs has not been elucidated [105]. These reports further highlight some known and potential crosstalk points between hypoxia and NF-κB. Emergence of new JmjC targets and functions may lead to new NF-κB regulatory links.

### 5.2. TAK and IKK in Hypoxia Induced NF-κB

An IKK independent mechanism of action was reported when hypoxic activation of NF-κB was initially discovered [106]. Since this seminal work, it has been shown that IKK dependent mechanisms of NF-κB activation in response to hypoxia do occur [72,107,108]. Our laboratory has shown that NF-κB responds rapidly to hypoxia in an IKK dependent manner in cancer and primary cell lines. Specifically, IKK mediates hypoxia induced phosphorylation of IκBα at serine 32 and 36 and also influences DNA binding of NF-κB [90,107]. This mechanism is dependent on the E2 ubiquitin conjugating enzyme Ubc13 [107], and XIAP may be one the E3 ligases interacting with Ubc13 in hypoxia-induced NF-κB activation [90]. We went on to show that hypoxia-induced IKK-mediated NF-κB activation is conserved in *Drosophila* [109]. This work also found that the MAPK family member TAK1 (Transforming Growth Factor-β-Activated Kinase 1) was part of the mechanism of hypoxia-induced NF-κB. Further investigation into other potential regulatory mechanisms of hypoxia induced NF-κB activation, particularly cell/tissue specific regulators, are required.

### 5.3. Role of IκBα in the Hypoxia Induction of NF-κB

As mentioned above, inactivation of the NF-κB inhibitor IκBα by TAK-IKK mediated serine phosphorylation can induce hypoxia-induced activation of NF-κB. Mutational analysis shows that this mechanism appears to be independent of IκBα tyrosine phosphorylation [107], for which a role was initially suggested [106]. Hypoxia induction of NF-κB is atypical since IκBα is not degraded as ubiquitination is inhibited and replaced with sumoylation in low oxygen environments [107]. There are currently various models of IκBα sumoylation influencing NF-κB activity in different cellular contexts [110,111]. Sumo 2/3 conjugation of IκBα may be important in hypoxia-induced NF-κB activation, whilst Sumo 1 conjugation has been shown to inhibit NF-κB. Inactivation of Sumo proteases in hypoxia is a potential mechanism whereby 2/3 conjugation of IκBα is present in hypoxia. Interestingly, polycomb complex regulated transcription has been shown to be influenced by nuclear IκBα phosphorylation and sumoylation [112]. Transcriptional control regulated by IκBα sumoylation in hypoxia represents a new area of research in the field of inflammation following hypoxia.

## 6. Hypoxia-Dependent NF-κB Activation in Cancer

### 6.1. Colorectal Cancer

Colorectal cancer (CRC) affects over 500,000 people each year, and is the fourth most common cause of cancer related mortalities [113]. The intestinal lumen of a CRC patient is characterised by both inflammatory and hypoxic regions (reviewed in [11]). NF-κB is activated in CRC in response to inflammation, promoting tumorigenesis and cancer progression [114]. Multiple pathways are implicated in NF-κB oncogenic role in CRC, including Reactive Oxygen Species (ROS) production, activation of pro-inflammatory cytokines, cell survival, EMT, cell proliferation, migration and angiogenesis (reviewed in [11]). Blocking NF-κB signalling has been shown to impair tumour growth in mouse models of CRC and CAC [40,115]. Moreover, anti-inflammatory drugs are used in the clinic to target chronic inflammation in CRC. These are mainly Non-Steroid Anti-Inflammatory Drugs (NSAIDs), which inhibit cyclooxygenase enzymes including COX2, upregulated by NF-κB. Like most solid tumours, hypoxia promotes tumorigenesis and progression in CRC. As mentioned earlier, effects of HIF-1α and HIF-2α on cancer is context specific. In CRC they have antagonising roles, with HIF-1α acting oncogenic and HIF-2α acting tumour suppressive [116]. High HIF-1α levels are associated with poor CRC prognosis. The role of HIF-1α in potentiating CRC through metastatic and angiogenic pathways has been characterised in several reports [117,118,119,120,121]. Conversely, transcript analysis on 120 CRC patient samples found that low HIF-2α mRNA is a prognostic factor, correlating with increased risk of mortality [122]. Another study performed immunohistochemically analysis on 63 primary tumour samples, finding an anti-correlation between HIF-2α levels and tumour stage [116]. The aforementioned study also used mouse xenografts showing siRNA depletion of HIF-1α reduces tumour growth whereas the opposite is seen in HIF-2α depletion. Additionally, HIF-1α knock-down in colon cancer cells increases cell proliferation, and, although HIF-2α has no effect on cell proliferation, colony formation was increased in a soft agar assay for anchorage independent growth [116]. Pharmacological inhibition of HIF has also been shown to result in tumour regression in a murine model of CAC, with a reduction in TAM infiltration [123]. As the above data demonstrates, NF-κB and HIF-1α are key components in driving CRC development and growth. Apoptosis, cell proliferation, angiogenesis and EMT are some overlapping pathways in the crosstalk between inflammatory and hypoxic signalling in CRC.

Around 70% of CRCs follow a distinct mutational sequence, starting with mutations in the tumour suppressor Adenomatous Polyposis Coli (APC) followed by V-Ki-ras2 Kirsten rat sarcoma viral oncogene homolog (KRAS), p53 and DCC (Deleted in Colorectal Cancer) mutations. The oncogene c-myc is also commonly overexpressed. The initial APC mutations trigger adenoma formation, which can develop into CRC [124]. APC supresses Wnt/β catenin signalling, which limits cell proliferation through the T-Cell Factor/Lymphoid Enhancer Factor (TCF/LEF) pathway [125,126]. APC, HIF-1α and β catenin are in cross regulatory network. APC is a HIF-1α target gene repressed in hypoxia, activating cell proliferation via increased Wnt/β catening signalling [127]. Moreover, APC can indirectly repress HIF-1α in a β catenin and NF-κB dependent fashion [127]. Furthermore, β catenin regulates NF-κB in a dose dependent manner. β catenin can activate NF-κB signalling through a positive feedback loop, however at higher levels β catenin can repress NF-κB (reviewed in [128]). β catenin levels have also been recently shown to be regulated by KDM2A and KDM2B [129]. Demethylation of non-phosphorylated β catenin by KDM2A and KDM2B induces nuclear degradation of β catenin and loss of Wnt/β catenin signalling. Given the importance of APC in CRC, gaining a better understanding of this complex crosstalk between HIF, NF-κB and APC pathways may give better insight into understanding molecular mechanisms behind CRC.

Another connection point between HIF and NF-κB pathways is c-myc. c-myc promotes cell proliferation and is typically overexpressed in transformed cells. HIF-1α inhibits c-myc activity and functioning via multiple mechanisms, including direct interaction, induction of Mx1 and activation of p21 [130,131,132,133]. Paradoxically, in an oncogenic environment where c-myc is overexpressed, HIF does not impair c-myc driven cell proliferation; instead, c-myc and HIF-1α collaborate to potentiate activation of metabolic proteins, such as Pyruvate Dehydrogenase Kinase 1 (PDK1) and Hexokinase 2 (HK2), driving the Warburg effect, and VEGF, driving angiogenesis (reviewed in [134]). c-myc is also transcriptionally upregulated by NF-κB, along with p21, conferring cell proliferative effects of NF-κB [16,135]. Furthermore, c-myc transcriptional activity on a subset of its targets driving cell growth is augmented by HIF-2α [136].

EMT is crucial for cancer progression, enabling invasion, and thus initiation of metastasis. The EMT driving transcription factors Snail and Twist are components co-regulated by NF-κB and HIF with potential clinical significance in CRC. Twist enhances EMT, Snail promotes lymph node metastasis in CRC [137]. Both are upregulated by hypoxia in a HIF dependent manner [138,139,140]. Also, HIF-1α upregulation of Twist in response to hypoxia or HIF-1α overexpression induce EMT and metastatic phenotypes [139]. TNF-α induction of NF-κB signalling, stabilising Snail and β catenin, also promotes EMT [141]. Moreover, high levels of Twist and NF-κB are associated with tumour metastasis to the lymph nodes [142]. Hypoxic and inflammatory stimuli in the tumour microenvironment can coordinate the infiltration TAMs to the tumour. TAMs can be tumour inhibitory or tumour promoting. Furthermore, low oxygen and chronic inflammation can subvert normal macrophage function from cancer killing (M1 classically activated) to cancer survival and growth (M2 alternatively activated). TAMs polarized towards the M2 state potentiate immunosuppressive, metastatic and angiogenic signals. NF-κB is activated by infiltrating macrophages and TAMs through release of growth factors and cytokines (reviewed in [143]), and is central to subverting TAM function. As mentioned earlier, pharmacological inhibition of HIF has also been shown to result in tumour regression in a murine model of CAC, with a reduction in TAMs infiltration [123]. The group identified a potential mechanism for the reduced TAM infiltration in this model through loss of Macrophage Colony Stimulating Factor Receptor (M-CSFR), a HIF target gene that is key signal for recruitment of macrophages to a tumour environment. The role played by TAMs in CRC progression is somewhat controversial. There is accumulating evidence for both tumour surviving and tumour killing phenotypes. It seems that which activity is dominant is dependent on where TAMs are located in CRC and the extent to which they have M1 or M2 like activities. Nonetheless the role of hypoxia and inflammation in tumour promoting functions of TAMs are well established. The development of therapies to polarize the TAM pool in CRC towards an M1 phenotype does seem attractive.

JmjC enzymes are emerging as new therapeutic targets and potential biomarkers in several cancers, including colon cancer. Given that KDM2A directly repressed NF-κB transcriptional activity via p65 demethylation [98], it would be anticipated that KDM2A activity may influence NF-κB driven cancer phenotypes. In the same study identifying KDM2A as demethylating p65, the authors showed that KDM2A impairs NF-κB dependent colon cancer cell growth [98]. Transcript analysis of colorectal liver metastases has identified the HIF-1α target KDM3A, as a biomarker for hypoxic tumour cells and potential prognostic marker and therapeutic target for CRC [69,144]. Another JmjC containing protein, KDM6B, is transcriptionally induced by vitamin D, and KDM6B mediates a subset of effects of vitamin D on colon cancer [145]. Specifically, KDM6B deletion induces the expression of pro EMT factors including Snail, and mesenchymal markers. KDM6B depletion was also shown to block vitamin D regulation of β catenin export. Moreover, KDM6B expression anti-correlates with Snail expression and correlates with vitamin D receptor expression [142].

PHD3 may function as a tumour suppressor in CRC through inhibition of IKKβ-mediated NF-κB signalling [146]. PHD3 has reduced expression in CRC compared to normal tissue and expression of PHD3 anti correlates with tumour severity [146]. Furthermore, PHD3 inhibits TNF-α induced NF-κB activity in colon cancer cell lines by blocking phosphorylation of IKKβ. This mechanism was shown to be independent of PHD3 hydroxylase activity, and via PHD3 blocking the interaction between IKKβ and Heat Shock Protein 90 (HSP90), an interaction required for IKKβ phosphorylation [146]. Control of p53 regulated apoptosis is another point of potential PHD-NF-κB crosstalk with clinical significance in CRC. PHD1 has been shown to aid the p53 mediated CRC resistance to genotoxic agents [147]. p53 phosphorylation, mediated by p38α kinase in response to chemotherapy, can inhibit chemotherapy-induced apoptosis through p53-induced DNA repair. Researchers found that silencing of PHD1, but not PHD2 or PHD3, prevented p53 activation in response to genotoxic treatment [147]. Moreover, PHD1 sensitized colorectal cancer to 5-FU treatment in mice [147]. Mechanistically, PHD1 was reported to enhance the p53-p38α kinase interaction, and subsequent p53 phosphorylation, in response to genotoxic damage in CRC in a hydroxylation-dependent and HIF-independent manner [147].

### 6.2. Breast and Lung Cancer

Breast and lung cancer are other examples of solid tumours where inflammation and hypoxia are fundamental drivers of disease progression. HIF and NF-κB signalling pathways have been established as playing oncogenic roles suitable for targeting. The usual players in inflammatory and hypoxic signalling crosstalk in cancer, such as TAMs, VEGF, and p53 have mechanistic importance in these diseases, as does the less well characterised crosstalk component, JmjC enzymes. In cell culture, IKK inhibition blocks Erb-B2 Receptor Tyrosine Kinase 2 (ERBB2) activation of NF-κB and induces apoptosis in ER negative ERBB2 positive breast cancer cells [148]. In vivo, inhibition of NF-κB impairs tumour progression in a murine model of breast cancer [148]. Furthermore, NF-κB dependent induction of EMT has been shown in breast cancer models [29,149]. There is also evidence of Epidermal Growth Factor (EGF) signalling enhancing NF-κB activity in breast cancer [150]. In this context, mechanism of activation of NF-κB by EGF may be similar to that described in lung cancer, where an IKK independent mechanism mediated by tyrosine kinase phosphorylation of IκBα is in place [151]. NF-κB activation is particularly prevalent in lung adenocarcinomas with constitutively active EGF-Receptor (EGFR) mutations. It can drive resistance of tumours to receptor tyrosine kinase inhibition, demonstrating the potential of targeting NF-κB to improve patient outcome in EGFR mutant lung cancer sufferers treated with receptor tyrosine kinase inhibitors [152]. An oncogenic role of HIF-1α in breast cancer has been well characterised (reviewed in [153]). Drugs inhibiting HIF, namely acriflavine, digoxin and topotecan impair tumour growth and metastasis in animal models of breast cancer [154,155,156]. Non-Small-Cell Lung Carcinoma (NSCLC) is highly metastatic and the most common form of lung cancer. HIF-1α and -2α subunits and frequently overexpressed in NSCLC, along with VEGF, which is targeted for treatment of NSCLC. HIF signalling has been shown to induce TAM mediated angiogenesis in human breast carcinoma models, and HIF-2α/TAM signalling may be a useful antiangiogenic breast cancer therapy [157,158]. Along with VEGF, Arginase 1 (Arg1) is transcriptionally induced by TAMs in response to HIF signalling. Arg1 also contributes to the tumour survival and growth activities of TAMs, and is elevated in mouse cancer model TAMs and in myeloid cells of breast cancer patients [159]. Several hypoxia-inducible HIF target JmjC enzymes are deregulated in breast and lung cancer. The KDM4 family members of JmjC enzymes, targeting di- and trimethylated H3K36 and H3K9, namely KDM4A, KDM4B and KDM4C are upregulated in breast cancer [160,161,162]. KDM4B has been found to mediate oestrogen stimulated cell proliferation of mammary cancer [163]. This study also found that KDM4B is transcriptionally induced by ERα in MCF7 cells and upregulates ERα target genes. Control of ERα target gene expression in mediated by fine tuning of H3K4 and H3K9 methylation at ER target promoters through a complex of KDM4B and the H3K4 methyltransferase MLL2 [163]. The H3K4 demethylase KDM5B has transcriptional repressor functions and is also a coactivator of the androgen receptor. KDM5B is overexpressed in both lung and breast cancer [164,165]. Furthermore, oncogenic functions of KDM5B have been demonstrated in vitro and in vivo. In MCF7 cells and in a mouse breast cancer model, depletion of KDM5B inhibits cell growth [166]. This correlates with suppression of the tumour suppressor gene BRCA1 [166]. More recently, high KDM5B expression was found to correlate with poor prognosis in breast cancer patients and enhanced breast cancer invasiveness in triple negative breast cancers [167]. Authors in this study also identified a mechanism for increased breast cancer invasion involving a KDM5B-metastasis-associated lung adenocarcinoma transcription (MALAT1)-hsa miR448 signalling axis [167]. A role of KDM5B is also present in lung cancer whereby KDM5B suppresses p53 expression [168]. This could have a functionally relevant link to NF-κB in lung cancer. Concomitant loss of p53 function and constitutively active KRAS control enhanced NF-κB activity in lung cancer cell lines and a mouse model of lung cancer [169]. Given the evidence for KMD5B driving cancer progression in the breast and lung cancers, it is perhaps no surprising that small molecule inhibitors against it are in development for use as potential cancer drugs. The small molecule inhibitor of KDM5B, EPT-103182 [170], has yielded promising results with anti-proliferative effects in various cancer cell lines and anti-tumour effects in mouse cancer models [171]. KDM2A along with KDM5B, have the highest frequency of gene amplifications and over expressions in breast cancer with respect to JmjC enzymes [172]. Interestingly the small isoform of KDM2A, which lacks the JmjC domain, is more highly expressed than the full isoform in a subset of breast cancer, suggesting an oncogenic role KDM2A independent of direct demethylase activity [172]. A potential mechanism for NSCLC progression in a subset of patients overexpressing KDM2A has been reported, with KDM2A stimulating cell proliferation through ERK1/2 signalling [173].

Studies on the importance of PHDs in breast and lung cancer are fairly limited. However, there is evidence of an important role of PHD1 in breast cancer through regulation of cell proliferation. Knockdown of PHD1 in breast cancer cell lines reduces cell proliferation and this correlates with loss of cyclin D1 [174]. Cyclin D1 stimulates cell cycle progression through its interaction with Cyclin Dependent Kinases (CDKs). Forkhead Box O3a (FOXO3a) was then identified as a new target for PHD1 [96]. Hydroxylation of FOXO3a by PHD1 has been shown to regulate cyclin D1 transcription, representing a potential mechanism by which PHD1 loss in breast cancer can impair cell growth. Conversely, treatment of breast cancer cell lines and a mouse xenograft model with docetaxel, a potential breast cancer drug, causes cell death in hypoxic conditions through c-Jun N-terminal Kinase 2 (JNK2)-PHD1 mediated HIF-1α degradation [175]. Interestingly, expression of individual PHD isoforms associates with good breast cancer patient outcome and PHD1 and PHD3 appear to be important in breast cancer in a HIF independent manner [176]. PHD3 is expressed highly in breast cancer patients with good prognosis and may be an important regulator of apoptosis in breast cancer [176]. PHD studies in the lung cancer are even more limited. Some groups have looked at the expression of PHD isoforms in lung cancer samples [177,178]. These studies show that PHDs are highly expressed in lung cancer compared to normal tissues, and both collective and individual PHD isoform expression are poor prognostic factors for NSCLC survival, independent of HIF levels [177,178]. As of yet, no clear mechanistic links between PHDs and NF-κB activity, independent of HIF, have been made in context of lung cancer and breast cancer.

## 7. Future Prospective in Cancer Therapeutics: Targeting HIF and the NF-κB Pathway

Due to the substantial contribution of HIF and NF-κB in carcinogenesis, over the years new therapeutic strategies have been developed to specifically target these two pathways. Moreover, considering their intimate crosstalk, it is possible that some of the therapeutics and modulators used might exert their function on both transcription factors. So far, HIF signalling has been altered mainly through PHD inhibitors, which have been beneficial in the treatment of several cancers, including those characterised by prominent inflammation. PHD inhibition can stabilise HIF-α, but also activate other pathways, such as NF-κB [72,91], promoting an inflammatory resolution. To date, five PHD inhibitors (BAY-853934, JTZ-951, FG-4592, AKB-6548 and GSK1278863) have entered clinical trials. However, some concerns about their side or off target effects exist, considering the different substrate specificity and cellular expression of the three enzymatic isoforms identified so far [179]. An alternative approach to target HIF stabilisation might be via the inhibition of the HIF-E3 ubiquitin ligase responsible for HIF degradation. Given that neddylation has been shown to modulate HIF via Cullin-2, MLN-4924, an adenosine monophosphate analog able to deneddylate cullin proteins, can act as a potent HIF stabilizer in vitro and in vivo, being also a viable tool in the treatment of cancer cells [180,181]. Additionally, VHL inhibitors represent an attractive alternative to PHD inhibitors. Recently, our group described VH298, a novel potent chemical probe blocking protein-protein interaction between VHL and HIF-α, downstream of HIF-α hydroxylation. Importantly, this small molecule highly selectively stabilises the hydroxylated form of HIF-1α and HIF-2α, in a concentration- and time-dependent manner, in both cancerous and non-cancerous primary cells, inducing a HIF-dependent hypoxic response [179]. On the other hand, several studies questioned the efficacy of a therapeutic activation of HIF, since this signaling pathway is directly involved in tumor promotion, as previously mentioned. Therefore, several other compounds are currently in clinical trials as HIF-1α inhibitors, although, in many cases, they were originally intended to target different pathways (i.e., PI3K/mTOR inhibitors, microtubules targeting agents, cardiac glycosides, topoisomerase inhibitors, among others) [182].

HIF-1α and HIF-2α have often divergent roles in tumorigenesis, hence the necessity to selectively target one or the other. AKB-4924 is a PHD inhibitor tested in inflammation models, showing a relative selectivity for HIF-1α versus HIF-2α [183]. However, no HIF-1α specific inhibitors have been discovered so far, although great efforts have been made to identify inhibitors exerting their function by decreasing mRNA or protein level of HIF-1α, preventing its dimerization or DNA/co-activators binding. For long time, HIF-2α has been considered undruggable, till the revolutionary discovery of two small molecules, PT2399 and the closely related analogue PT2385. These compounds function as potent HIF-2α antagonists, being able to bind to a large cavity located in the PAS-B domain of HIF-2α, thus disrupting the hetero-dimerization between HIF-2α and HIF-1β [184,185]. These small molecules have a promising clinical potential, considering that they reduced tumour growth and decreased tumour vascular area in VHL^−/−^ clear cell Renal Cell Carcinoma (ccRCC) patient derived xenographs, showing even greater efficacy than conventional treatments (i.e., sunitinib) [184,185,186].

As previously explained, not only HIF, but also NF-κB is a key player in many aspects of cancer development and progression. For this reason, the possibility to target directly NF-κB for cancer therapy has been an important subject of research. On one side, due to the deep link between inflammation and cancer, it would be ideal to prevent or treat tumour formation by blocking inflammation [44]. On the other side, in the light of the multiple functions of NF-κB in the innate and adaptive immune responses, the use of NF-κB inhibitors would not be recommended to treat malignancies, especially in the tumour-eliminating phase, when immune cells specifically target transformed cells. Nevertheless, using chemotherapeutics in combination with inhibitors of NF-κB seems to be currently the preferred approach, in particular when tumours feature chronic inflammation [5]. Many well-known NSAIDs, such as aspirin and ibuprofen, can be used to this scope. In fact, at low doses, aspirin has been suggested to prevent some types of cancer, including colorectal cancer [187], whereas at high doses, aspirin can inhibit the kinase activity of IKK or interfere with the degradation of IκBα, blocking NF-κB activity. Alongside NSAIDs, anti-inflammatory and anti-cancer activities have been recognised for a number of natural products, glucocorticoids, immunosuppressants, or inhibitors of other pathways, directly affecting the NF-κB induction or signalling cascade, the translocation of NF-κB to the nucleus, the DNA binding of the dimers or their interactions with the transcriptional machinery [3].

The use of NF-κB inhibitors has been shown to be important also to enhance the activity of immunotherapy. In a recent study, a combined use of IL-18 as immunotherapeutic agent alongside a targeted inhibition of the NF-κB pathway emerged as potentially effective against pancreatic cancer [188]. Recently, cancer immunotherapy effectiveness has been boosted by a better understanding of immune checkpoints. Programmed Death 1 (PD1)/Programmed Death Ligand 1 (PD-L1) and Cytotoxic T-Lymphocyte Associated Protein 4 (CTLA-4) are the most critical immune checkpoints. Both of them are used by tumours to escape host immune surveillance, although they have distinct expression and mechanisms of action in the regulation of T cell activity [189]. Given the importance of the hypoxic tumour microenvironment for TAMs and tumour-infiltrating myeloid cells, it is not surprising that PD-L1 was identified as a direct HIF-1α target gene in myeloid-derived suppressor cells (MDSCs) [190]. Several reports correlated a high expression of PD-L1 to a poor prognosis in a number of solid cancers. Thus, immune checkpoint blockade with monoclonal antibodies directed against PD-1 and PD-L1 has proven to be powerful compared to conventional chemotherapies, activating an anti-tumour immunity able to recognise specifically tumour derived antigens, even when they have undergone mutations [189]. Several PD1 and PD-L1 antibodies are currently in clinical trials (reviewed in [189]), representing surely the next generation of the immune modulators. However, whether HIF and NF-κB crosstalk extends to immunosuppression has yet to be formally investigated and surely is an area of extreme importance.

NF-κB signalling is upregulated in cells having a compromised expression of the tumour suppressor pVHL, such as ccRCC cells, where VHL is inactivated [191]. In this cellular context, Vascular Cell Adhesion Molecule 1 (VCAM-1) expression was found to be regulated by the non-canonical NF-κB pathway. Importantly, VCAM-1 decreased following VHL loss or after hypoxia exposure and PHD inactivation [192]. Recently, the mechanisms by which pVHL might directly impact on the NF-κB pathways have been proposed. Wang and colleagues demonstrated that pVHL mediates the K63-ubiquitination of IKKβ. Surprisingly, this modification does not lead to degradation, but prevents TAK1-IKKβ interaction, and consequent IKKβ phosphorylation and NF-κB activation [193]. Considering this novel function of pVHL regulating the NF-κB pathway, new therapeutic possibilities might be speculated, especially to inhibit the aberrant activation of the NF-κB pathway in some neoplastic contexts. Therefore, further studies are needed in this promising direction, as the main challenge for researchers in this field is still to directly target NF-κB, as well as HIF, only in transformed cells.

## 8. Conclusions

NF-κB and HIF crosstalk occurs at many levels, from shared activators to shared target genes. However, context specificity exists and this is an important determinant in whether this crosstalk can be used for future therapeutic intervention. Since, pharmacological interventions for both pathways are available, future studies investigating the role of HIF modulators on the NF-κB pathway are underway with the aim of broadening the use of these compounds in the clinic. Furthermore, the identification of novel compounds targeting specific transcription factor dimers such as HIF-2α-HIF-1β also opens the possibility of specifically targeting NF-κB dimers in the future.
